# Substrate Neutrality for Obtaining Block Copolymer Vertical Orientation

**DOI:** 10.3390/polym16121740

**Published:** 2024-06-19

**Authors:** Kaitlyn Hillery, Nayanathara Hendeniya, Shaghayegh Abtahi, Caden Chittick, Boyce Chang

**Affiliations:** Department of Materials Science and Engineering, Iowa State University, Ames, IA 50011, USA

**Keywords:** block copolymer, self-assembly, directed self-assembly, lithography

## Abstract

Nanopatterning methods utilizing block copolymer (BCP) self-assembly are attractive for semiconductor fabrication due to their molecular precision and high resolution. Grafted polymer brushes play a crucial role in providing a neutral surface conducive for the orientational control of BCPs. These brushes create a non-preferential substrate, allowing wetting of the distinct chemistries from each block of the BCP. This vertically aligns the BCP self-assembled lattice to create patterns that are useful for semiconductor nanofabrication. In this review, we aim to explore various methods used to tune the substrate and BCP interface toward a neutral template. This review takes a historical perspective on the polymer brush methods developed to achieve substrate neutrality. We divide the approaches into copolymer and blended homopolymer methods. Early attempts to obtain neutral substrates utilized end-grafted random copolymers that consisted of monomers from each block. This evolved into side-group-grafted chains, cross-linked mats, and block cooligomer brushes. Amidst the augmentation of the chain architecture, homopolymer blends were developed as a facile method where polymer chains with each chemistry were mixed and grafted onto the substrate. This was largely believed to be challenging due to the macrophase separation of the chemically incompatible chains. However, innovative methods such as sequential grafting and BCP compatibilizers were utilized to circumvent this problem. The advantages and challenges of each method are discussed in the context of neutrality and feasibility.

## 1. Introduction

**Figure 1:**
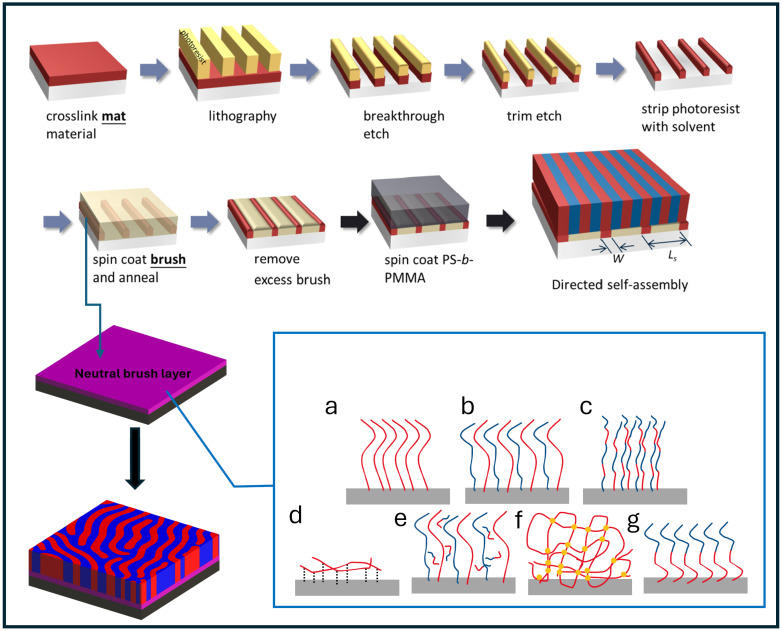
Schematic of nanofabrication process of chemically patterned substrates [29] highlighting the direct assembly of a BCP process using various forms of neutrality: (**a**) homopolymer brushes; (**b**) mixed homopolymer brushes; (**c**) random copolymer brushes; (**d**) side-chain brushes; (**e**) ternary homopolymer brushes; (**f**) cross-linked polymer mats; (**g**) block cooligomer brushes. Colors represent polymers with distinct repeat units. Adapted with permission from references [1,29].

Thin films of block copolymers (BCPs) are emerging as an energy-efficient and precise method for high-resolution nanopatterning. Periodic structures are self-assembled through the microphase segregation of chemically distinct blocks. Depending on their Flory–Huggins interaction parameter (χ), their segmental length (N), and the volume fraction (ϕ) of each block, both the lattice structure and periodicity (L_0_) are thermodynamically fixed [1,2,3,4,5,6]. It is precisely this molecular definition that has attracted the application of BCP self-assembly to complement extreme ultraviolet lithography (EUV) to rectify pattern stochastics [7]. BCPs have also been applied to improve resolution—a method known as density multiplication—where aggressive scaling (<30 nm) has been achieved from the tried and tested 193 nm immersion lithography (193i) [8,9]. However, the use of BCPs for patterning requires orientational control using a templated substrate, a process formally known as directed self-assembly (DSA). The BCPs are subjected to either thermal or solvent annealing or a combination of both for the lattice structure to propagate on the template [10,11,12]. The templated substrate is critical to DSA and it serves two purposes: (i) vertical orientation, where phase-segregated blocks face the top surface (the central theme of this review); and (ii) alignment of the lattice along the substrate for long-range order [1,2,5,13]. Downstream, DSA sets the line-space (lamellar) or contact-hole (cylinder) pattern normal to the surface, allowing top-down selective etching processes that leverage the distinct chemistry of each block for pattern transfer into the substrate. A general process of this patterning method is outlined in Figure 1.

The key to achieving vertical orientation in BCPs is the use of a chemically neutral surface towards all blocks, meaning no preference in any of the blocks to wet the surface. This neutrality requirement applies to both the polymer–substrate and polymer–air interfaces. Thus, the challenge of obtaining vertical patterns can be attributed to the mixed chemistry of the BCP, in which one block generally demonstrates a preference toward a particular interface; hence, it dominates wetting. Interfacial energy minimization forces the orientation of the lattice to be parallel to the substrate. For vertical orientation to occur, a critical window must be defined to prevent bias from either blocks of the BCP, meaning their interfacial mismatch is roughly equal or “neutral” at the substrate and the polymer–air interface. For example, in the case of polystyrene-*block*-poly(methyl methacrylate) (PS-*b*-PMMA) on a silicon substrate, the PMMA block expresses a higher preference toward the silicon oxide than its counterpart, PS [14,15,16]. Thus, surface modification is necessary to neutralize the preference for a vertical BCP alignment with respect to the substrate.

Several methods have been examined to obtain orientational control such as electric fields [17], solvent annealing [11,18,19], topologically roughened surface treatment [20], and neutralizing the interface [13,21,22,23,24,25,26]. Most commonly, surface neutralization is achieved using random copolymers (RCPs). PS-*r*-PMMA brushes in various forms were first introduced in a seminal work by Mansky et al. to mitigate the BCP’s surface preference [13]. It is important to note that here that film thickness is limited because the influence of the surface diminishes with increasing volume. The BCP film thickness should not significantly exceed the characteristic period, L_0_, to ensure orientation is controlled via interfacial energy at the substrate or air interface. 

In this review, we discuss various surface-neutralizing methods employed for BCP orientational control and outline the progression of neutral-surface methods. Although we acknowledge the significance of a top coat for neutrality at the polymer–air interface, especially in high-χ BCPs [27,28], this review focuses on the efforts regarding the polymer–substrate interface. In general, the methods involve mixing the chemistry of both blocks to create an effectively neutral substrate. This is achieved using copolymers or homopolymer blends. The progress of both routes is compared, and unique opportunities in homopolymer blending are highlighted. Finally, we discuss emerging methods of controlling surface chemistry in the context of BCP orientation control.

## 2. Early Developments in Obtaining BCP Self-Assembly

Graphoepitaxy utilizes confinement in the form of topographical features such as trenches to achieve orientational alignment and represents some of the earliest demonstrations of substrate neutrality. Here, BCP chains are oriented by (i) the chemical modification of the side and bottom walls, and (ii) the commensurability of the trench size with its periodicity, forcing alignment in order to maximize conformational entropy. Although this method can be highly successful when obtaining ordered BCPs, it also poses limitations such as chemical stability, overfilling and underfilling of BCP trenches, high costs, and interference with further processing if the structures cannot be removed [30,31,32]. For these reasons, chemoepitaxy methods are highly advantageous to maximize feature density [9]. Rather than topography, chemoepitaxy primarily relies on sparse chemical patterns to guide the orientation of the BCP lattice. Here, a neutral surface is utilized as a spacer between guiding patterns and chemistry preferentially wets one of the blocks. Ideally, BCP chains above the neutral surface adopt the orientation of the “guided” chains through careful control of their commensurability to the BCP period.

Prior to modern DSA methods, BCP alignment through neutral interfaces has been investigated since the 1990s. In particular, confining lamellar-forming BCP films between two hard parallel walls was a well-investigated method to understand the effects of confinement on the BCP’s bulk interactions, phase segregation, natural period, and surface interactions [33,34,35]. It was understood that thickness played a significant role in obtaining symmetric and antisymmetric films, and incommensurate unconfined films were accommodated via the step height (L_0_) at the free surface where both blocks could be present at both interfaces, as identified from works by Bates and Russell et al. [36,37,38]. Confining the films via rigid supports with the preferential wetting of one of the blocks resulted in parallel orientation, despite incommensurate spacing with the L_0_ of the BCP, which frustrated the polymer with enthalpic and entropic penalties in free energy. To this effect, Kellogg et al. were motivated to isolate the effects of these frustrations; hence, they reduced preferential adsorption by applying random copolymers (RCPs) composed of the BCP block components to the rigid wall [33]. In obtaining perpendicular lamellae, they demonstrated the importance of the non-biased neutrality required for the BCP to relax into perpendicular domains with respect to the interface. This gave rise to the uptake of RCP usage as an inexpensive way to orient diblock copolymers by controlling the interfacial energy preference toward either block in the BCP. However, this method posed limitations such as that unanchored RCPs may have exhibited mixing with the BCP layer and unremitting processes. This, in turn, gave rise to other work to fill these gaps. This limited the effect the RCPs could have on the BCP by not allowing the localization of the RCP to influence the self-assembly behavior. 

**Figure 2:**
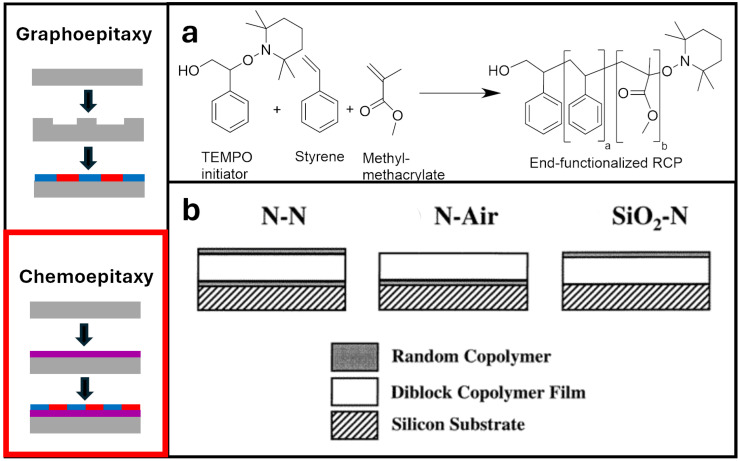
Left: Simplified schematics of directed self-assembly routes. Colors represent polymers with distinct repeat units. Right: (**a**) Nitroxide-mediated radical polymerization for hydroxyl-terminated end-functionalized RCPs inspired by Mansky’s study. (**b**) Alternative confinement of a BCP thin-film using RCP derivatives [39]. Adapted with permission from reference [39].

Further mixing between the RCP and thin-film BCP was alleviated by Mansky et al., who demonstrated end-grafting of the RCP onto a substrate to control interfacial interactions between the BCP film and the substrate. Furthermore, by controlling the composition of the monomer input of the RCP before synthesis, they were able to alter the neutrality of the RCP brushes toward a lamellar-forming BCP (Figure 2a). It was found that roughly sixty percent styrene was needed in the pre-synthetic composition for non-preferential RCPs, which were then functionalized and anchored to the substrate. There was also little to no preferential segregation; thus, the resulting BCP successfully self-assembled into lamellae patterns. This led to the following works by Huang and Mays, which encased the orientation of the copolymer microdomains normal to the surface by altering both the surface and air interfaces [24]. This was accomplished by incorporating previous works that had demonstrated that perfluoroalkyl-terminated polymers immigrated toward the air interface and that had used hydroxy-terminated RCPs for the surface interface. Figure 2b displays a schematic of these alterations. By adapting Mansky’s anchored RCPs and adding surface-activated RCPs to the top, they demonstrated sufficient neutrality coverage on either interface of the BCP [39]. However, this may be redundant for BCPs with a low χ such as PS-*b*-PMMA because later findings showed that the top surface had near-equal interfacial energies at elevated temperatures. Hence, the BCP orientation may be sufficiently adaptable by modifying one interface. 

## 3. Alternative Approaches to Obtaining Neutrality Using Copolymers

**Figure 3:**
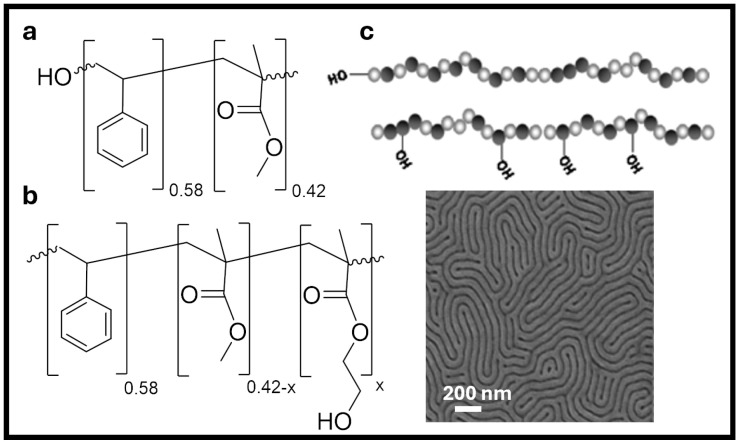
PS and PMMA RCPs of (**a**) end-hydroxyl functionalized brush, (**b**) side-chain hydroxyl-containing brush, (**c**) schematic of (**a**) and (**b**), respectively, based on the work of Nealey and Gopalan, followed by an SEM image of BCP of PS-*b*-PMMA (52-*b*-52) atop a side-chain brush of f_St_ = 0.58, f_MMA_ = 0.41, and f_HEMA_ = 0.01 [40]. Adapted with permission from reference [40].

Since the recent uptake in the use of RCP brushes, researchers have naturally explored other avenues to modify these systems to alleviate known limitations. Thus, Nealey and Gopalan et al. explored the use of grafting RCPs from side-chain anchoring rather than end-grafting polymers, as displayed in Figure 3a [40]. This was accomplished by the addition of 2-hydroxyethyl methacrylate (HEMA) during polymerization to form the hydroxyl-containing side-group series at various ratios (Figure 3b). The role of polydispersity (PDI) on grafted brushes was also slightly explored using classical and living synthetic routes to obtain polymers with differing PDIs. Yet, with multiple covalent binding sites now introduced to the polymer, the effect of examining PDI was rendered obsolete compared with adjusting the precursor ratios of monomers. Overall, substrate neutrality was achieved (Figure 3c). The primary improvement from this approach was faster kinetic binding than end-grafted RCPs due to the increased number of grafting sites. The changes in chain conformation due to the multiple sites, however, led to a shallower brush thickness and lower graft density, which has been shown to negatively impact neutrality due to the lack of “proximity shielding” of the substrate akin to self-assembled monolayers [41,42].

**Figure 4:**
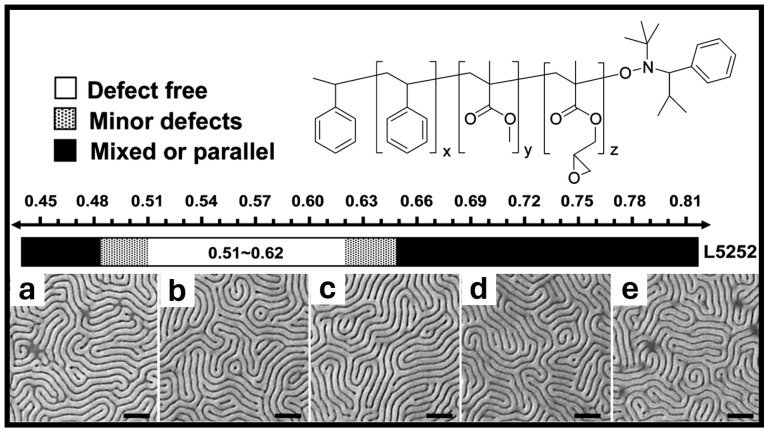
Epoxy-containing RCP for use as cross-linking neutral mat with a schematic of achieved neutrality of BCP of PS-*b*-PMMA (52-*b*-52) atop the cross-linked neutral mat. f_GMA_ = 0.01 for all images and varies by styrene fractions such that: (**a**) f_St_ = 0.48, (**b**) f_St_ = 0.53, (**c**) f_St_ = 0.56, (**d**) f_St_ = 0.59, and (**e**) f_St_ = 0.63, scale bar represents 200 nm [43]. Adapted with permission from reference [43].

Within a few years, Han, Nealey, and Gopalan et al. introduced an extensive technique utilizing cross-linking thin-film mats (Figure 4) [26,43]. This study utilized the range of neutrality from RCP brushes as previously outlined by Mansky et al. [13]. These cross-linkable RCPs were used as a neutral template for BCP orientational control by altering the PS fractions to PMMA fractions and keeping the cross-linking fraction of glycidyl methacrylate (GMA) constant. They successfully demonstrated the formation of perpendicular domains of BCP using cross-linked RCP mats within a given neutrality window (Figure 4a–e). These mats were suggested to be more chemically stable toward resisting materials compared with polymer brushes [2], thus providing improved reproducibility, which is critical for high-volume manufacturing. We limited our discussion on cross-linkable RCPs due to the comprehensive reviews available elsewhere [2,16].

**Figure 5:**
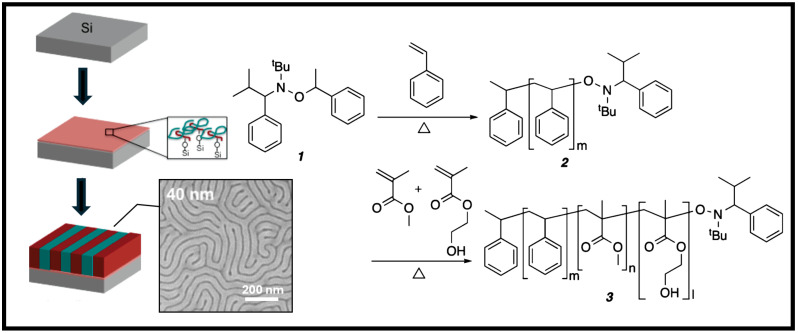
(Left) Block cooligomer brushes (1.6–2.5 kg/mol) of f_St_ = 0.64 grafted onto the substrate prior to annealing the BCP of PS-*b*-PMMA (52-*b*-52) atop. (Right) Nitroxide-mediated polymerization of O(S-*b*-M*r*H) block cooligomer [21]. Adapted with permission from reference [21].

A few years later, Ji and Nealey’s study emerged, aiming to alleviate this tunable window crisis by acknowledging that the synthesis of an RCP may be highly problematic for polymer systems other than PS and PMMA [21]. This approach aimed to create a simpler means of creating a neutral surface by preparing a block cooligomer in sequence for a monomer addition. Using low molecular weight (1.6–2.5 kg/mol) blocks of f(st) = 51% and f(st) = 64%, with an average of 1.5 HEMA units per chain, they demonstrated a successful neutrality for BCP self-assembly, as displayed in Figure 5. This study highlighted the use of polymer brushes as a neutral surface without employing RCPs as long as the polymer brush blocks were sufficiently smaller than the BCP blocks to ensure mixing. 

Other methods of providing neutrality have been investigated without the use of RCPs. Efforts here have focused on using homopolymer blends as a simpler approach for neutrality, if macrophase separation could be mitigated. In the following sections, we discuss the literature, focusing on non-RCP neutrality. Additionally, we provide a brief history of how early research influenced the field, the grafting processes to mitigate homopolymer separation, and the scope and limitations when obtaining a neutral surface from homopolymer brushes.

## 4. A history of Homopolymer Blends

**Figure 6:**
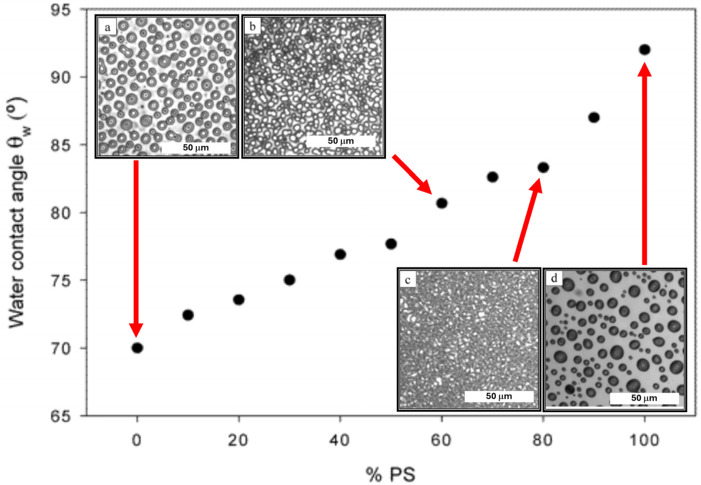
Water contact angle measurements of hydroxy-terminated PS (Mn = 3.8 kg/mol) and PMMA (Mn = 4.4 kg/mol) as a function of PS composition with corresponding optical microscopy images of 80 nm thick film with equal weights of PS = 22 kg/mol and PMMA = 23 kg/mol annealed atop PS and PMMA brushes with compositions (**a**) 100% PMMA-OH, (**b**) 60% PS, (**c**) 80% PS, and (**d**) 100% PS [44]. Adapted with permission from reference [44].

Winesett and Ade et al. were among the earliest to report tuning substrate neutrality through the blending of homopolymer brushes [44]. End-hydroxyl-terminated PS and PMMA were grafted as a blend onto a Si substrate. The random grafting of both chemistries was assumed, despite a preference for PMMA to wet the substrate [33]. Nonetheless, their water contact angle (WCA) measurements agreed with this conflicting assumption and showed a proportional increase in WCA as PS within the blend increased (Figure 6). It was further claimed that the results were similar to the work previously outlined by Mansky et al. using RCP brushes. Note that a linear trend between the WCA and composition is to be expected for RCPs due to their fixed composition. This is a critical difference from mixed homopolymers because, as previously discussed, it is known that PMMA has a higher preference toward the silicon substrate, and these interfacial energy differences would be evident in practice. Therefore, it is unlikely that a linear WCA trend from 0 to 100% PS blend would be observed. Due to the preference of PMMA, the WCA behavior should exhibit overwhelming PMMA characteristics up to a point where a saturated PS is able to overcome the interfacial preference [14,15]. Rather than using BCPs, films of a 1:1 PS–PMMA homopolymer blend were used to test for neutrality. Here, the lack of macrophase separation was attributed to a neutral substrate at 80% and 90% PS. In contradiction to their claims, the neutrality window deviated from Mansky et al., which observed neutrality from RCPs at 60% PS [24].

Despite achieving neutrality in the homopolymer blend, this study was not cited as a successful attempt at gaining neutrality by the BCP community, perhaps due to the absence of tests using BCP films (speculative). Thus, many groups have gravitated toward employing modifications to avoid brush phase separation to arrive at substrate neutrality [14,15,45].

**Figure 7:**
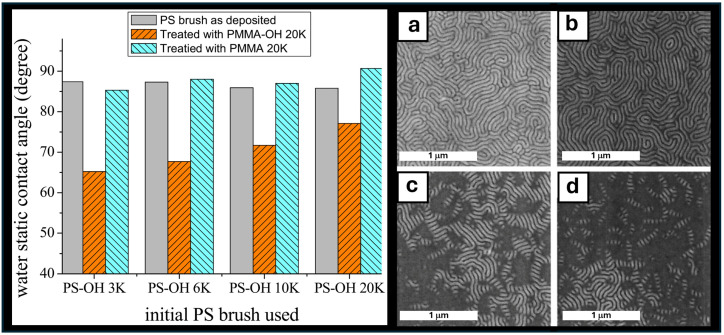
(Left) Water contact angles of PS brushes before modification (grey), after PMMA-OH 20 (kg/mol) insertion (orange), and PS brushes treated with PMMA 20 (20 kg/mol) (teal). (Right) SEM images of BCP PS-*b*-PMMA (52-*b*-52) annealed on the inserted brushes of PMMA in PS with the relative Mn as follows: PS:PMMA (kg/mol) (**a**) 3:20, (**b**) 6:20, (**c**) 9:20, and (**d**) 20:20 [14]. Adapted with permission from reference [14].

One study from Liu et al. demonstrated a two-step method using PS and PMMA brushes to investigate the wetting properties of PS polymer brushes by PMMA insertion [14]. Firstly, they outlined that PMMA-OH bound to the substrate, forming a brush layer using the PS brushes. This was carried out by employing a control of non-functionalized PMMA with the same annealing and washing process. This can be briefly observed in the chart displayed in Figure 7. They also examined the effect of a reverse order of insertion and found that when PS-OH (3 kg/mol) was inserted into PMMA-OH (20 kg/mol), the grafting percentage was found to be around 9.4% PS and 90.6% PMMA. Alternatively, when PMMA-OH (20 kg/mol) was inserted into PS-OH (3 kg/mol), the composition was approximately 50.4% PS and 49.6% PMMA, further demonstrating the interfacial preference of PMMA to the surface. Overall, this demonstrated that mixed homopolymers, when grafted in sequence, could grant access to a neutral surface for BCP self-assembly.

**Figure 8:**
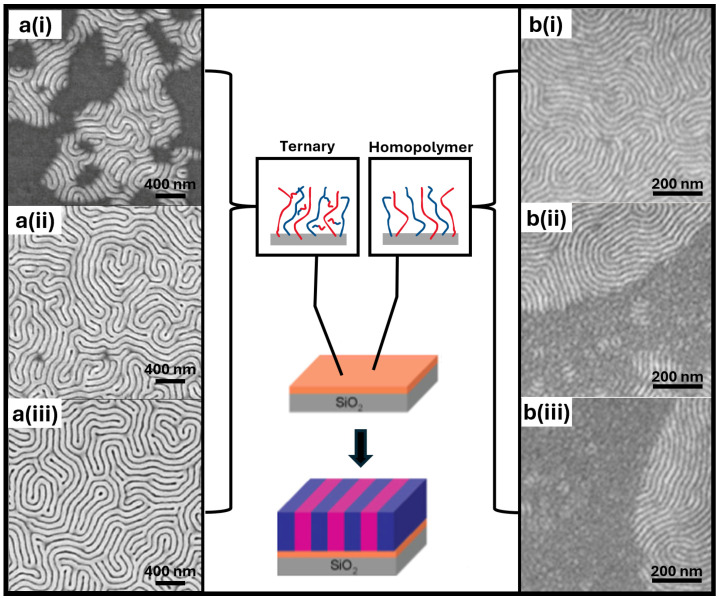
(**a**) SEM images of self-assembled PS-*b*-PMMA (52k-*b*-52k) on homopolymer brushes made from a blend solution (1 wt%) containing 70% BCP blender (5k-*b*-5k) and 30% homopolymers of PS-OH (6 k) and PMMA-OH (6 k) before rinsing. The following images display these ratios of the 30% homopolymer brushes of PS:PMMA: (**i**) 6:4, (**ii**) 5:5, and (**iii**) 4:6 [23]. (**b**) SEM images of PS-*b*-PMMA (50k-*b*-50k) on long-chain binary homopolymer-blend brushes grafted without a BCP blender using PS-OH (16 kg/mol) and PMMA-OH (15 kg/mol). The following images represent these ratios of the cast blend PS:PMMA: (**i**) 85:15, (**ii**) 80:20, and (**iii**) 75:25 [15]. Adapted with permission from references [15,23].

Another study carried out by Ji and Nealey et al. prepared homopolymer brushes using blends of hydroxyl-terminated homopolymers with a low molecular weight BCP; in this case, it was around ten percent of the thin-film BCP size. The BCP “compatibilizer” had to have a significantly low molecular weight so as not to microphase segregate, but assist the homopolymer in mixing. After grafting the homopolymers, this “blender BCP” was removed from the surface along with non-grafted excess chains before a larger thin BCP film was applied; the selected data are shown in Figure 8a(i–iii). Thus, the blender BCP did not interfere with the self-assembling BCP film. As previously observed by other works, PMMA generally has a higher affinity for silicon substrates than PS. However, with the addition of a blender BCP, defect-free vertical lamellae were obtained, despite a homopolymer brush composition of 40% PS-60% PMMA and being near defect-free at a ratio of 30% PS-70% PMMA. Many blends were reported to have more defects and were unsuitable as a neutral template [23]. Interestingly, the authors reported that neutral substrates were unattainable in the absence of BCP compatibilizers.

Nearly a decade later, Ceresoli and Sparnicci et al. similarly utilized homopolymers to gain neutrality; however, in direct contradiction to Ji and Nealey et al., they did not employ a blending agent. Instead, they employed long-chain homopolymers of PS-OH (16 kg/mol) and PMMA-OH (15 kg/mol) in the hope of gaining a thicker brush with minimal separation for a neutral template, in contrast to Ji and Nealey’s 6 kg/mol brushes [15]. However, the window of neutrality of lamellae-forming BPC atop the long-chain brushes was reported to be severely restricted. Although pockets of neutrality were achieved, there was a largely homogenous texture from non-neutral conditions that dominated when the PS content was less than 85% (Figure 8b(i–iii)). The reported composition of the grafted brush after rinsing was determined using a unique approach of the TGA-GC-MS chromatographic relative area of the styrene and methyl methacrylate with respect to the corresponding mass per charge (*m*/*z*). The neutrality achieved here challenged the preconceived notions and warrants a revisiting of homopolymer-blended brushes. Areas of opportunity here include balancing grafting characteristics, blend morphology, and asymmetric brush structures.

Shortly after, Pang and Ji et al. developed a single homopolymer approach where a brush or mat with monomers that were chemically distinct from both blocks of the BCP were used as a neutral substrate [46]. They successfully prepared vertically oriented BCP films of PS-b-PMMA, poly(styrene-b-rac-lactide) (PS-b-PDLLA), and poly(styrene-b-propylene carbonate) (PS-b-PPC). Although this method eliminated the need for mixed chemistry (either copolymerization or blending), the identification and selection of homopolymers for ideal neutrality can be challenging. Furthermore, it was demonstrated that small changes in the monomer structure could lead to a loss of neutrality.

## 5. Surface Characterization

**Figure 9:**
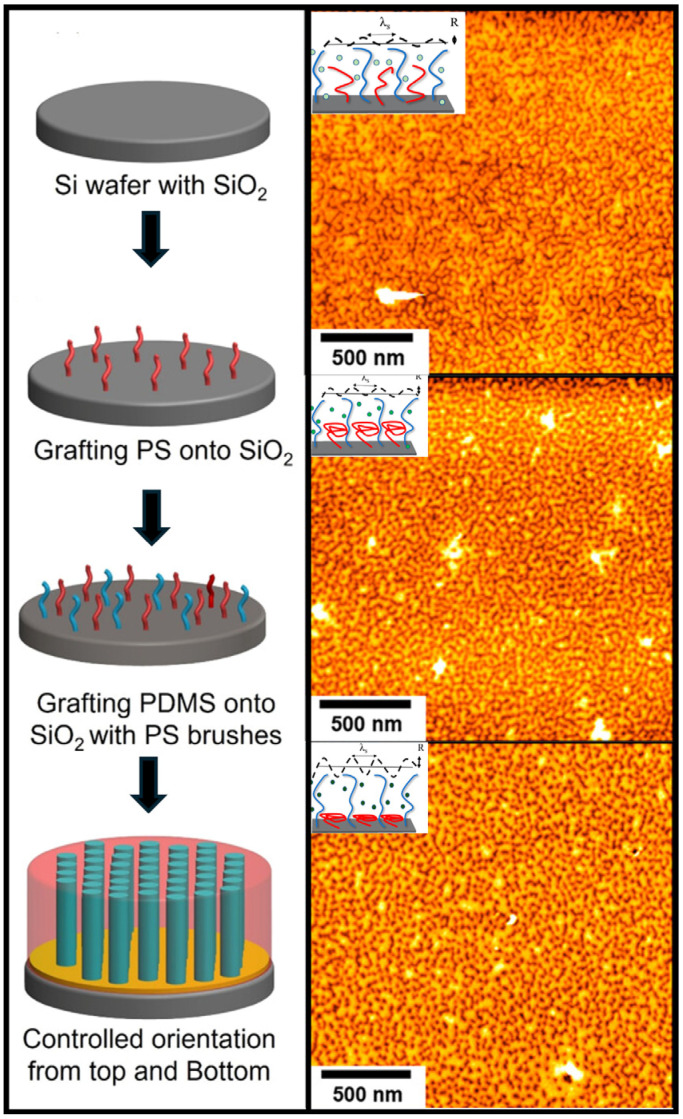
(Left): Schematic of two-step insertion process of homopolymers for a neutral surface using PS-OH (17.4 kg/mol) and PDMS-OH (17.8 kg/mol) to orient cylinder-forming BCPs: PS-b-PDMS [45]. (Right): Tapping mode SPM topographical images of PS-OH (71.6% surface composition) and PDMS-OH (28.4% surface composition) with schematics depicting expected brush and amplitude fluctuations. (Top) is a weak PS-selective solvent, (middle) is a moderately PS-selective solvent, and (bottom) is a highly PS-selective solvent [68]. Adapted with permission from reference [68].

The surface characterization of polymer brushes is vital to understand the underlying chemical and mechanical properties. Non-invasive characterization techniques such as atomic force microscopy (AFM) [47,48,49], photo-induced force microscopy (PiFM) [50], angle-resolved XPS, and NEXAF are a few techniques that are often used. AFM and PiFM are scanning probe techniques. This involves mounting a sharp tip on a three-dimensional scanning device of subatomic precision [51]. Soft materials such as polymers and polymer brushes are commonly imaged using the tapping mode, where the van der Waals interactions define the attractive/repulsive forces and, therefore, contrast. AFM can image both dry [52] and wet [47] brushes over a length-scale varying from nanometers to micrometers. Macroscopic phase separation and morphology are provided as a function of graft density [53,54,55]. Polymer brushes comprise stimuli-responsiveness, and AFM can be a highly successful technique in characterizing and monitoring their behavior in different environments [56,57]. For example, the topography changes initiated by reorganizing polymer brushes under various solvent exposures can be captured using AFM (Figure 9). PiFM, however, is more beneficial when probing the chemical structure. It provides an alternative to diffraction-limited traditional Fourier transform infrared spectroscopy (FTIR), which is unable to resolve sub-10 nm molecular arrangements [58]. PiFM is essentially AFM equipped with an optical system to probe molecular resonance. It has the ability to spatiochemically image specific domains [50]. Polymer blends do not usually have the ability to respond to resonant Raman enhancement where long-signal integration is required [58]. Therefore, the mechanical detection of molecular resonance offered by PiFM is uniquely positioned to understand the chemical properties of polymer brush blends. 

Additionally, near-edge X-ray absorption fine structure (NEXAFS) spectroscopy can be a powerful technique to analyze the electronic and structural properties of ultrathin molecular layers by non-destructively revealing both the structure and chemistry of thin organic films. This technique quantifies the density of bonds involving elements such as carbon, nitrogen, oxygen, and fluorine; develops depth profiles; and determines bond orientation. By measuring the absorption of linearly polarized soft X-rays near the K-shell threshold, NEXAFS provides an element-selective analysis of low-Z elements, making it particularly effective when studying molecular structures at interfaces [59]. It offers detailed surface composition profiles from the top 1 to 6 nanometers of the material, which is crucial for understanding both surface and near-surface chemistry in polymer brushes. By measuring the electron yield at different kinetic energies corresponding with various depths within the material, NEXAFS offers a detailed depth profile of the chemical composition by applying a variable negative voltage bias to the detector grid, selectively detecting electrons emitted from specific depths [60]. 

This offers advantages over X-ray photoelectron spectroscopy (XPS) and ultraviolet photoelectron spectroscopy (UPS) by focusing on unoccupied states and providing complementary information rather than examining occupied states in the core and valence regions. Additionally, NEXAFS is highly sensitive to chemical changes and the chemical environment of atoms, allowing the detection of subtle variations in materials that might be convoluted XPS and UPS [61]. Understanding the distribution of components such as photo-acid generators (PAGs) within photoresist is essential for successful photolithography [62]. A significant issue NEXAFS addresses is the segregation of small-molecule additives such as PAGs at the surface of the polymer brush during processing. This segregation can alter the surface chemistry, impacting the performance and functionality of the polymer brush. For instance, in chemically amplified photoresists, the presence of PAGs at the surface can affect the interfacial photoresist structure, composition, and deprotection kinetics, leading to problems such as T-topping and closure [60,62]. Another concern for polymer brushes is accurate thickness determination. Optical methods such as ellipsometry [63,64] and reflectometry [65,66] techniques are typically employed for film thicknesses ranging from tens to hundreds of nanometers. In contrast, angle-resolved XPS is extensively utilized to study very thin layers, typically only a few nanometers thick [67].

## 6. Outlook and Future Work

The bulk of the literature discussed here focuses on PS-b-PMMA as a model system. Although this provides valuable insights, PS-b-PMMA is unable to scale below a 20 nm pitch for lithography due to its low χ [69]. Therefore, the methods for neutrality developed for this system need to be adapted into high-χ BCPs such as PS-*b*-PDMS (polydimethylsiloxane) and PS-*b*-P2VP (poly(2-vinyl pyridine)). However, synthesizing random copolymers from chemically incompatible monomers (high χ) is challenging in practice. The mixed homopolymer brush approach was applied to PS-*b*-PDMS for orientational control utilizing the two-step insertion method outlined by Liu, Nealey, and Himpsel [14,45,68]. Panda and Ho et al. demonstrated their efforts to gain control of cylinder-forming BCPs using PS and PDMS homopolymer brushes. Figure 9 displays a generalized schematic of this approach, achieving surface control. By sequentially grafting long-chain homopolymers, they effectively achieved well-ordered perpendicular PDMS [45]. In addition, a similar work further investigated the topography of PS and PDMS homopolymer brushes and examined the swelling and contracting behavior of the chains with a cylinder-forming BCP capped with a neutral top layer [68]. This was reported using solvent vapor annealing (SVA) with PS-selective solvents and determined the amplitude of the roughness based on the coil and stretching of the brushes when subjected to various solvents. This is an interesting concept, where the chemical environment influenced the organization of the homopolymer brushes, leading to topographical transformations. For example, amplitude fluctuations (R)—given values of a lower amplitude corresponding with a weak PS-selective solvent, a moderately PS-selective solvent, and a highly PS-selective solvent—were reported to be R = 1.72 ± 0.55, R = 2.89 ± 0.73, and R = 2.98 ± 0.61, respectively. Aside from the successful demonstration of film-spanning perpendicular cylinders via PS-b-PMMA thin-films, this work demonstrated that surface-responsive brushes could be significant for the DSA of high-χ BCPs.

In addition, synthetic strategies could be used to circumvent the challenges of creating neutral brushes for high-χ BCPs. A promising approach is utilizing polymerization techniques that exhibit a highly asymmetric reaction ratio of monomers (such as surface-initiated atom transfer radical polymerization) to obtain alternating copolymers [70]. This technique has been applied to the preparation of polyelectrolyte brushes (PEBs) with tunable surface energy. The PEBs exhibited unique properties such as repulsive electrostatic and steric interactions due to a charged group in the repeating polymer chain [70]. Additionally, the responsiveness of PEB to factors such as the pH, salt, solvent, and counterions could be augmented based on the comonomer.

## 7. Conclusions

In conclusion, many studies have successfully demonstrated the alteration of a substrate using polymer brushes over the last three decades. In this review, we have discussed previous work that paved the way for substrate tuning in BCP self-assembly methods, from the confinement of the BCP to RCP brush control via controlling synthetic monomer ratios to variations in these species, including cross-linked polymer mats, side-anchored brushes, and block cooligomers. We have outlined the brushes not RCP in nature, which still expressed neutrality for orientational control, as well as the widely adaptable two-step insertion method of mixed homopolymer brushes and the various attempts to overcome interfacial energy mismatch, including adjusting the polymer molecular weight and adding a blending agent to relax phase separation. Finally, we have discussed alternative methods derived from previous works to branch out into new approaches and applications such as modifying cylinder-forming BCPs and using alternating polymer systems. These studies provide simple templates to obtain neutrality and tunability, and offer insights for the future development of BCP nanopatterning.
